# Effects of zinc supplementation and implant abscess on the immune system and growth performance of growing beef steers

**DOI:** 10.1093/tas/txae075

**Published:** 2024-05-02

**Authors:** Emma Rients, Carlos Franco, Fabian Diaz, Jodi McGill, Stephanie Hansen

**Affiliations:** Department of Animal Science, Iowa State University, Ames, Iowa, USA; Department of Veterinary Microbiology and Preventive Medicine, Iowa State University, Ames, Iowa, USA; Department of Veterinary Microbiology and Preventive Medicine, Iowa State University, Ames, Iowa, USA; Department of Veterinary Microbiology and Preventive Medicine, Iowa State University, Ames, Iowa, USA; Department of Animal Science, Iowa State University, Ames, Iowa, USA

**Keywords:** cattle, immune system, implant abscess, zinc

## Abstract

Seventy-two Angus-cross steers (261 ± 14 kg) were utilized to determine the effects of supplemental Zn sulfate on growth, trace mineral status, circulating immune cells, and functional innate immune responses. Steers were stratified by weight and implanted with a Component E-S with Tylan implant (Elanco Animal Health, Greenfield, IN) on day 0. Dietary treatments included: control (CON; no supplemental Zn), Zn100 (100 mg supplemental Zn/kg DM), and Zn150 (150 mg supplemental Zn/kg DM). Analyzed dietary concentrations of Zn were 58, 160, and 207 mg Zn/kg DM, respectively. On days 13 and 57, blood from nine steers per treatment was collected for immune analyses (cell phenotyping and response to stimulus). On day 16, implant abscesses were evaluated by palpation and visual appraisal. Sixty percent of steers had abscesses; however, there were no differences in abscess prevalence due to treatment (*P* = 0.67). Data were analyzed as a split-plot design using the Mixed procedure of SAS 9.4 (Cary, NC) with effects of dietary treatment, abscess, and their interaction. There was a tendency (treatment × abscess; *P* ≤ 0.09) for steers without abscesses to have greater average daily gain (ADG; treatment × abscess *P* = 0.06) and gain:feed (G:F; treatment × abscess *P* = 0.09) from d 14 to 27 in CON and Zn100 while within Zn150 steers without abscesses tended to have lesser ADG and G:F than abscessed steers. There were no other treatment × abscess effects for growth performance, but steers with abscesses tended to have decreased final body weight (*P* = 0.10) and overall G:F (days 0 to 57; *P* = 0.08). There was no interaction of treatment and abscess on immune cell populations on days 13 or 58 (treatment × abscess *P* ≥ 0.11). On day 13, Zn150 steers had increased CD45RO + gamma delta (*P* = 0.04) T cells. Abscessed steers had increased CD21 + B cells (*P* = 0.03) and tended to have increased CD21 + (*P* = 0.07) and CD21 + MHCII^hi^ (*P* = 0.07) B cells in circulation. This study shows zinc supplementation and implant abscesses can alter the immune system and growth performance of growing beef steers.

## Teaser

This study investigates the interaction of dietary zinc, growth, and the immune system in cattle while also examining the effects of implant abscesses. The findings highlight the importance of minimizing implant abscesses to optimize the health and performance of feedlot cattle.

## Introduction

Zinc supplementation above NASEM (2016) recommendations can support increased growth with growth-promoting technologies such as beta-agonists and implants (Genther-Schroeder et al., 2016; Messersmith and Hansen, 2021). Young, growing cattle may also need additional Zn to support growth and immune response. Kegley et al. (2001) and Smerchek et al. (2023) observed changes in the immune system when supplementing Zn at higher than NASEM (2016) recommendations. Cattle seem to respond positively to increased Zn supplementation, and while the study by NASEM (2016) recommendations may prevent deficiency, they may be less than needed for optimizing growth and health in feedlot cattle.

While not in the original design of the study, 60% of steers developed abscesses at the site of anabolic implant insertion, creating an opportunity to study their effects on growth and immune function Previous studies have observed decreases in average daily gain (ADG) in cattle with implant abnormalities compared to cattle with normal implants (Bryant et al., 1999; Spire et al., 1999). Others have reported little or no difference in the growth performance of abscessed and non-abscessed steers unless the implant was not retained in the ear (Berry et al., 2000). With over 90% of cattle in US feedlots receiving at least one implant (APHIS, 2013), and the decreased performance observed with abnormalities, and abscessed ears due to implants has ramifications for the beef industry.

This study aimed to determine if supplementing Zn to growing calves would affect growth performance and immune response. The hypothesis is that dietary Zn supplementation will positively influence the growth performance and immune response in vitro assays in growing beef steers, with further investigation into how the presence of ear abscesses may interact with these effects.

## Materials and Methods

This study was approved by the Iowa State Animal Care and Use Committee (#21 to 150).

### Animals and Sample Collection

Two hundred steers purchased directly from a single ranch in southern Iowa were transported to the Iowa State Beef Nutrition Farm (Ames, IA). On arrival, steers were offered long-stem hay tops dressed with a common receiving total mixed ration (TMR). Steers were weighed, sorted by ideal body weight (BW) for the study, and given new electronic identification tags. Eighty-four steers were selected as potential animals for this study and were moved to pens (3.7 × 12.2 m) equipped with a GrowSafe bunk (Vytelle, Lenexa, KS) for 12 d prior to trial initiation. Each steer was equipped with a radio frequency tag that relayed individual steer feed disappearance data from the bunk to the GrowSafe software, therefore individual intake data were recorded for each animal in each pen. On day −1, steers were vaccinated for respiratory disease (Bovilis Vista 5 SQ, Merck Animal Health, Rahway, NJ). On day 0, 72 steers (261 ± 14 kg) were selected and stratified by initial BW into pens of six steers. Steers were administered a Component E-S with Tylan implant (Elanco Animal Health, Greenfield, IN) on day 0 on the backside of the ear according to the manufacturer’s instructions. Wet and rainy weather conditions on day 0 likely contributed to high incidence of ear abscesses at the implant site. Dietary treatments consisting of control (CON) with no supplemental zinc, Zn100 with 100 mg supplemental Zn/kg DM, and Zn150 with 150 mg supplemental Zn/kg DM added to the diet were randomly assigned. Zinc was supplemented as ZnSO_4_. Diets are shown in Table 1. Treatments were delivered as part of the TMR, with dried distiller grains plus solubles as a carrier. Steers were fed daily at approximately 0800 hours daily using a wagon mixer. Loads of feed were fed in increasing concentrations of Zn. The weight of feed in the bunk was recorded daily, and feed delivery was targeted to keep approximately 10 kg of feed in the bunk prior to the next feed delivery. Feed deliveries were recorded daily. Cattle had ad libitum access to water. Weekly TMR samples were taken and frozen until dried in a 70 °C oven for 48 h. Samples were ground, composited, and analyzed at a commercial laboratory (Dairyland INC, Arcadia, WI) for nutrient analysis (methods 990.03 and 920.39; AOAC, 1995). Individual steer dry matter intake (DMI) was calculated as fed intakes corrected for the dry matter of weekly TMR samples.

**Table 1.** Diet composition and nutrient analysis

Ingredient, % DM basis	CON	Zn100	Zn150
Corn silage	40	40	40
SweetBran	40	40	40
DDGS^*^	18.06	18.03	18.02
Limestone	1.5	1.5	1.5
Salt	0.31	0.31	0.31
Vitamin A and E premix^*^	0.1	0.1	0.1
Rumensin 90	0.0135	0.0135	0.0135
Trace mineral^*^	0.016	0.016	0.016
Zinc sulfate^*^	—	0.028	0.042
Analyzed composition			
DM, %	58.7	58.6	57.8
Crude protein^†^, %	18.89	19.01	18.60
NDF^†^, %	33.43	31.29	32.37
Ether extract^†^, %	5.05	4.87	4.86
Zinc, mg/kg DM	58	160	207

^*^DDGS were used as a carrier for vitamin and mineral and treatment premixes^.^ Vitamin and mineral premix provided vitamins and minerals (besides Zn) at 2016 NASEM recommendations. Treatment premixes provided 100 and 150 mg Zn/Kg DM, respectively^.^

^†^Values determined by Dairyland Laboratories (Arcadia, WI).

BW was collected on days −1, 0, 12, 13, 27, 28, 56, and 57. The average of 2-d BW measurements was used for analysis. Steers were locked back from feed prior to weighing and a 4% shrink was applied to all weights to account for gut fill. Liver samples (*n* = 18/treatment) were collected on days −7, 14, and 58 using methods described by Engle and Spears (2000). Briefly, the rib space between the 11th and 12th was shaved and scrubbed three times with betadine and 70% ethanol. An incision was made on a line from the hip bone to the point of the shoulder. A modified bone marrow biopsy needle (T-Handle Jamshidi Bone marrow needle, Becton, Dickinson and Company, Franklin Lakes, NJ), was inserted and suction was applied with a 12 mL syringe to collect liver. The sample was rinsed with cold phosphate-buffered saline (PBS; pH = 7) to remove blood and non-liver tissue and was stored on ice until transported to the lab. Liver was stored at −20 °C prior to trace mineral analysis. On days 12, 13, 56, and 57, blood was collected via jugular venipuncture for fresh blood immune cell assays (heparin; *n* = 18/treatment) with half of each treatment being collected on consecutive days. On days −1, 13, 28, and 57 blood was collected via jugular venipuncture from all steers (K_2_EDTA, heparin, and serum), centrifuged at 1,000 × *g* for 20 min, aliquoted and stored at −20 or −80 °C for further analysis.

On day 14, multiple steers were identified to have abscesses on their ears where implants were administered. A trained evaluator scored (Elanco Animal Health) abscesses on day 16 and meloxicam (285 mg) was administered to all steers to decrease pain and inflammation due to abscesses.

### Trace Mineral Concentrations

Plasma, liver, and composited TMR samples were analyzed for trace minerals using inductively coupled plasma-optical emissions spectroscopy (ICP-OES; Optima 7,000; PerkinElmer, Waltham, MA) using methods described in Pogge and Hansen (2013). Quality control samples (serum UTAK, Valencia, CA; bovine liver from National Institutes of Standards and Technology, Gaithersburg, MD) were included in all runs to verify instrument accuracy.

### Fresh Blood Analysis

Whole blood was utilized for three fresh blood, functional assays including pHrodo phagocytosis, dihydrorhodamine 123 reactive oxygen species (DHR), and stimulation on days 12, 13, 56, and 57. In pHrodo phagocytosis and DHR assays, cells were lysed using red blood cell lysing buffer before plating. Whole blood was also used for the determination of circulating frequencies of immune cell populations as described by Mahmoud et al. (2020). Stimulation of whole blood for cytokine secretion was also completed using methods described by Mahmoud et al. (2020) and McDonald et al. (2021).

The pHrodo phagocytosis assay was performed per the manufacturers instruction (Invitrogen, ThermoFisher Scientific). Briefly, cells were resuspended at 10^6^ in live imaging solution (Invitrogen, ThermoFisher Scientific) and plated in a 96-well culture plate. Cells were incubated for 90 min at 37 °C with 20 ug/well pHrodo E. Coli green bioparticles (Invitrogen, ThermoFisher Scientific). Cells were washed twice and then surface stained as described below, followed by flow cytometry analysis.

The DHR assay was completed as in the study by Mahmoud et al. (2020). Briefly, cells were resuspended in PBS and plated into a 96-well culture plate. The plate was spun and the supernatant was removed. Cells were resuspended in Hanks Balanced Salt Solution with DHR containing calcium chloride, magnesium chloride, and magnesium sulfate. The plate was incubated for 20 min at 37 °C, then phorbol myristate acetate was added to experimental stimulation wells and incubated for 25 min at 37 °C. The reaction was stopped by incubating the plate on an ice slurry for 5 min prior to being spun down, the supernatant removed, and cells resuspended in PBS.

Cells for both pHrodo phagocytosis and DHR assays were then stained as in the study by Mahmoud et al. (2020) with mouse-anti bovine CD14 (clone CAM36A, Kingfisher Biotechnology) and mouse anti-bovine granulocyte antibody (clone CH138A). Cells were washed and stained with anti-mouse IgG1-AF647 (Invitrogen, Life Science) and anti-mouse IgM-PE. Cells were fixed in BD FACS lysing buffer, washed with PBS then resuspended in PBS, and read using a flow cytometer at the Iowa State University Flow Cytometry Facility (BD FACS Canto; Ames, IA). Samples were analyzed using FlowJo software v10.7 (Benton, Dickinson & Company). Some samples from days 12 to 13 had errors in flow cytometry analysis and were excluded from the study.

## Statistical Analysis

The Glimmix procedure of SAS 9.4 was used to analyze the presence of abscesses within each treatment as binary data. Growth performance and blood measures were analyzed using the Mixed procedure of SAS. 9.4 as a split-plot design with steer as the experimental unit. The model included the effects of dietary treatment (TRT), the presence of abscess, and their interaction. Random intercepts were included for the steer within treatment groups using an unstructured covariance matrix. To allow for the exploration of negative variance components, the “nobound” option was utilized. Data from fresh blood assays on days 12, 13, 56, and 57 were analyzed together and will be referred to days 13 and 57, respectively for the remainder of the manuscript. Data from the fresh blood assays (pHrodo phagocytosis and DHR) and stimulations were log-transformed for analysis. Initial BW served as a covariate in growth performance data analysis. Initial liver mineral concentrations were used as a covariate in subsequent liver mineral analysis. Plasma trace mineral was analyzed as repeated measures for the effects of treatment, abscess, and day with initial plasma trace mineral values used as a covariate and the variance components covariance structure. If the interaction between treatment, abscess, and day was *P* ≥ 0.2, the model was simplified. Least squares mean (LSMEANS) were calculated for the interactions with pairwise differences examined for all possible interactions with significance determined as *P* ≤ 0.05. For log-transformed data, means and SEMs were back-transformed in Microsoft Excel (Redmond, WA). Outliers were determined on an individual steer basis with data points greater than three standard deviations away from the treatment mean for that particular parameter removed from analysis. Significance was determined as *P* ≤ 0.05 and tendencies were declared when 0.05 < *P* ≤ 0.1.

## Results

There was no difference between treatments in abscess incidence (*P* = 0.67).

### Growth Performance

There was a tendency for a TRT × abscess effect for ADG days 14 to 28 (*P* = 0.06; Table 2), where Zn150 steers with abscesses tended to have the greatest growth and CON and Zn100 steers with abscesses tended to have the lowest daily growth with all non-abscessed steers being intermediate and not different from all treatments. There also was a tendency for increased feed efficiency (G:F) in Zn150 steers with abscesses compared to CON steers without abscesses (TRT × abscess *P* = 0.09), when all other steers were intermediate and not different from all treatments. There were no other interactions between TRT and abscess for growth performance (*P* ≥ 0.13).

**Table 2.** Effects of dietary Zn supplementation and implant abscess on growth performance measures of growing steers

Dietary treatments^*^	CON		Zn100		Zn150			*P* Values		
Implant abscess^†^	No	Yes	No	Yes	No	Yes	SEM	Dietary TRT	Abscess	TRT × abscess
*n* (steers)	11	13	8	16	9	15				
*Body weight, kg*										
Day 0	261	—	262	—	261	—	2.99	0.97	—	—
Day 13^‡^^,^^||^	292	290	292	286	291	287	2.69	0.44	0.038	0.64
Day 27^‡^^,^^||^	321	317	326	313	321	323	5.05	0.79	0.146	0.20
Day 57^‡^^,^^||^	370	366	379	360	376	372	7.64	0.60	0.096	0.38
*ADG, kg*										
0 to 13^‡^	2.40	2.27	2.35	1.90	2.33	1.99	0.2073	0.44	0.04	0.64
14 to 28^‡^	2.07^xy^	1.77^y^	2.29^xy^	1.86^y^	1.96^xy^	2.37^x^	0.2197	0.40	0.49	0.06
29 to 57^‡^	1.68	1.67	1.82	1.62	1.89	1.70	0.1388	0.56	0.18	0.65
0 to 57^‡^	1.90	1.83	2.07	1.73	2.01	1.94	0.1340	0.60	0.09	0.38
*DMI, kg*										
0 to 13^‡^	7.3	7.2	7.4	7.4	7.5	7.1	0.4736	0.89	0.58	0.86
14 to 28^‡^	7.8	8.3	8.6	7.8	8.3	8.3	0.5642	0.84	0.87	0.32
29 to 57^‡^	7.9	8.8	9.3	8.6	8.5	8.7	0.5984	0.52	0.78	0.29
0 to 57^‡^	7.7	8.3	8.6	8.2	8.1	8.3	0.5311	0.70	0.74	0.52
*G:F* ^$^										
0 to 13^‡^	0.295	0.318	0.351	0.256	0.298	0.295	0.03725	0.95	0.34	0.14
14 to 28^‡^	0.278^xy^	0.212^y^	0.274^xy^	0.222^xy^	0.240^xy^	0.284^x^	0.03125	0.79	0.25	0.09
29 to 57^‡^	0.215	0.187	0.203	0.187	0.270	0.205	0.03033	0.19	0.09	0.62
0 to 57^‡^	0.252	0.218	0.246	0.210	0.293	0.245	0.03094	0.25	0.08	0.96

^*^CON = No supplemental Zn; Zn100 = 100 mg Zn/kg DM supplemental; Zn150 = 150 mg Zn/kg DM supplemental; Zn100 and Zn150 supplemented as ZnSO_4_.

^†^All steers were implanted with Component E-S with Tylan (Elanco Animal Health, Greenfield, IN) on day 0. Implant abscess status was determined by a trained evaluator (Elanco Animal Health) on day 16.

^‡^Day 0 body weight was used as a covariate for analysis.

^||^4% shrink applied to weight to account for gut fill.

^$^Gain:feed; Feed efficiency.

^x,y,z^within row, treatment means with different superscripts tend to differ 0.05 <* P* ≤ 0.10 from pairwise comparison.

Although there were no main effects of TRT (*P* ≥ 0.19) on DMI or growth, abscess influenced growth performance. On day 13, BW was greater in non-abscessed steers (*P* = 0.04) and on day 57 BW tended to be increased in non-abscessed steers (*P* = 0.10). Similarly, ADG was greater from days 0 to 13 for non-abscessed steers (*P* = 0.04), and tended to be greater in non-abscessed steers throughout the entire study (days 0 to 57; *P* = 0.10). Additionally, G:F for the entire study (days 0 to 57) tended to be greater in non-abscessed steers (*P* = 0.08). There were no abscess effects on DMI (*P* ≥ 0.58).

### Trace Mineral Concentrations

Liver Mn tended to be increased in CON steers with abscesses compared to Zn100 steers without abscesses on day 14 (TRT × abscess *P* = 0.07; Table 3). There were no other TRT × abscess interactions for liver trace mineral concentrations (TRT × abscess *P* ≥ 0.11). There was however a main effect of Zn treatment on liver Cu concentrations at the end of the study, where CON steers had increased liver Cu compared to Zn100 and Zn150 steers (TRT *P* = 0.04). There were no other main effects of TRT or abscess on liver trace mineral concentrations (*P* ≥ 0.12).

**Table 3.** Effects of dietary Zn supplementation and implant abscess on liver trace mineral concentrations

Dietary treatments^*^	CON		Zn100		Zn150			*P* Values		
Implant abscess^†^	No	Yes	No	Yes	No	Yes	SEM	Dietary TRT	Abscess	TRT × abscess
*n* (steers)	8	10	5	13	7	11				
*Cu, mg/kg DM*										
Day −7	214^b^		306^a^		251^ab^		24.7	0.04		
Day 14^‡^	282	261	253	242	247	272	17.2	0.21	0.82	0.17
Day 58^‡^	296	251	226	227	233	241	24.1	0.04	0.42	0.26
*Fe, mg/kg DM*										
Day −7	207		201		180		15.8	0.45		
Day 14^‡^	143	164	135	159	163	161	17.8	0.55	0.20	0.59
Day 58^‡^	129	123	137	150	146	135	11.3	0.12	0.78	0.38
*Mn, mg/kg DM*										
Day −7	7.0		7.0		6.3		0.31	0.25		
Day 14^‡^	7.4^xy^	8.2^x^	8.4^xy^	7.0^y^	7.8^xy^	7.6^xy^	0.62	0.94	0.54	0.07
Day 58^‡^	8.2	7.9	8.0	7.1	7.6	8.2	0.63	0.48	0.64	0.26
Zn, mg/kg DM										
Day −7	169		155		175		16.2	0.68		
Day 14^‡^	158	127	129	146	144	157	16.1	0.58	1.00	0.10
Day 58^‡^	132	106	113	133	142	122	20.7	0.49	0.68	0.31

^*^CON = No supplemental Zn; Zn100 = 100 mg Zn/kg DM supplemental; Zn150 = 150 mg Zn/kg DM supplemental; Zn100 and Zn150 supplemented as ZnSO_4_.

^†^All steers were implanted with Component E-S with Tylan (Elanco Animal Health, Greenfield, IN) on day 0. Implant abscess status was determined by a trained evaluator (Elanco Animal Health) on day 16.

^‡^Analyzed with day −7 as a covariate.

^a, b^within row, unlike superscripts, indicate differences between treatment means *P* ≤ 0.05 from pairwise comparison.

^x, y^within row, treatment means with different superscripts tend to differ 0.05 <* P* ≤ 0.10 from pairwise comparison.

**Figure 1.**
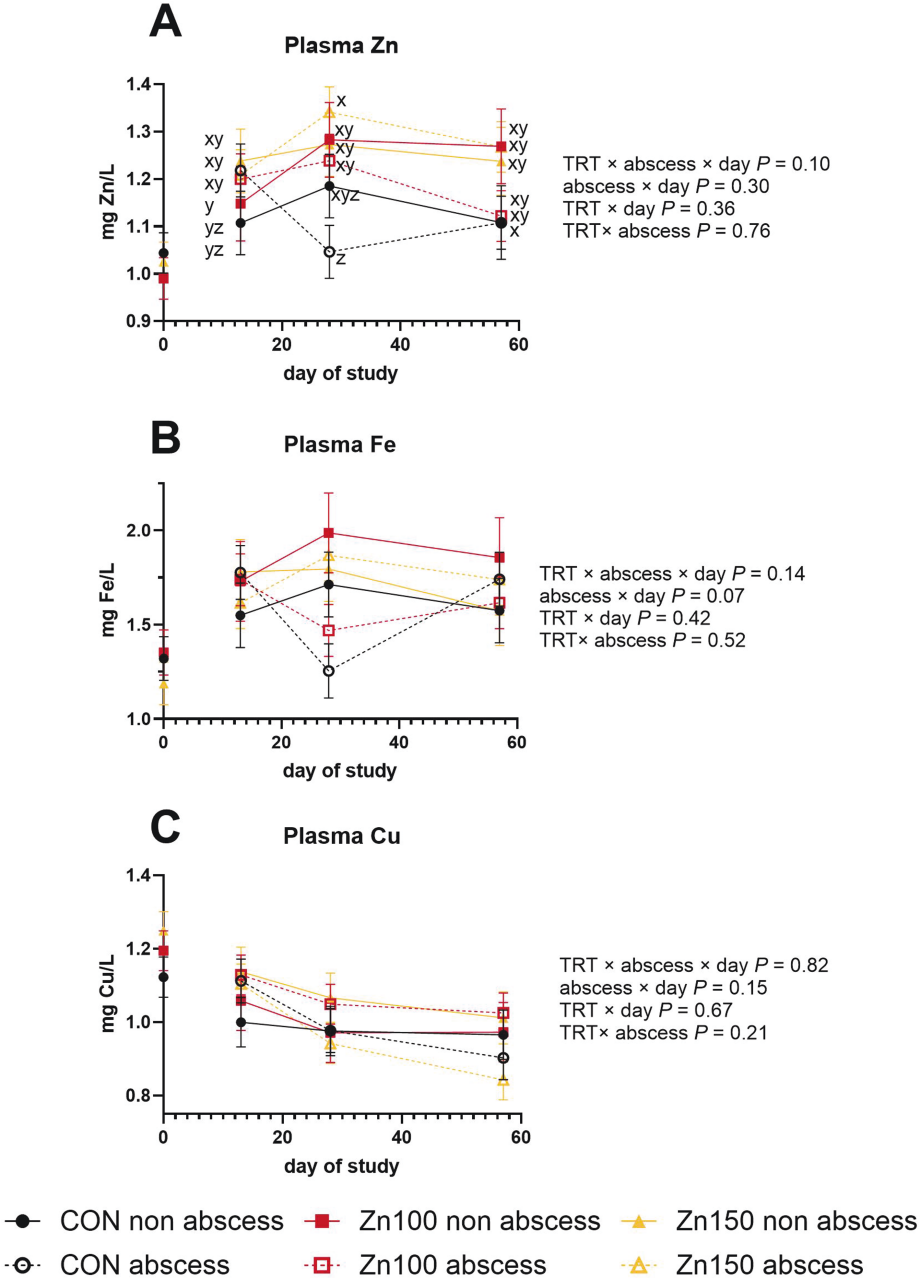
Steers were supplemented with no supplemental Zn (diet analyzed 58 mg Zn/kg DM), 100 mg Zn/kg DM (Zn100), or 150 mg Zn/kg DM (Zn150). All steers were implanted with a Component E-S with Tylan implant (Elanco Animal Health) and dietary treatments started on day 0. All steers were evaluated for implant abscesses on day 16 by a trained evaluator (Elanco Animal Health). Plasma was collected on days −1, 13, 28, and 57 for trace mineral concentration analysis. Data were analyzed as a split-plot design using repeated measures with day −1 used as a covariate in the mixed procedure of SAS. Unlike superscripts indicate a difference in treatments across timepoints (*P* ≤ 0.05). (A) Plasma Zn concentration tended to be influenced by treatment, abscess, and day (TRT × abscess × day *P* = 0.10). (B) Plasma Fe concentrations tended to be influenced by abscess and day (Abscess × day *P* = 0.07). (C) Plasma Cu was not influenced by the interaction of treatment, abscess and day, two-way interactions, or abscesses or treatment alone (*P* ≥ 0.15) but decreased over the study (day *P* < 0.01).

There was a tendency for a TRT × abscess × day interaction for plasma Zn (TRT × abscess × day *P* = 0.10; Figure 1) where on day 13, there are no differences between treatments, but on day 28 Zn150 with abscesses is greatest and CON with abscesses have the lowest. By day 57 CON with abscesses do not differ from CON non-abscess, which are lesser than Zn100 non-abscess and Zn150.

There was no interaction between TRT, abscess, and day for plasma Fe (*P* = 0.14). There was a tendency for a day-by-abscess effect (*P* = 0.07) where plasma Fe was similar on day 13, but abscessed steers tended to be decreased compared to non-abscessed on day 28, then returned to the same concentration as day 13 on day 57. There were no other interactions or main effects for plasma Fe (*P* ≥ 0.40).

There were no interactions between TRT, day, and abscess for plasma Cu (*P* ≥ 0.13). There was a main effect of day, where plasma Cu was highest on day 13 and was lower on days 28 and 58 (day *P* < 0.01).

### Immune Cell Populations

There were no TRT × abscess effects on day 13 immune cell phenotypes (*P* ≥ 0.26; Table 4). There were no TRT × abscess effects on day 56 immune cell phenotypes (*P* ≥ 0.11; Table 5).

**Table 4.** Effects of dietary Zn supplementation and implant abscess on circulating immune cell populations and immune response after 13 d

Dietary treatments^*^	CON		Zn100		Zn150			*P* Values		
Implant abscess^†^	No	Yes	No	Yes	No	Yes	SEM	Dietary TRT	Abscess	TRT × abscess
*n* (steers)^‡^	5	4	2	7	5	4				
*Cell populations, %* ^||^										
CD 21+	14.7	23.1	18.8	21.8	21.3	23.9	3.38	0.28	0.03	0.40
CD 21 + MHCII^hi^	13.1	18.4	17.8	19.6	19.6	21.5	3.16	0.10	0.12	0.67
CD5+	42.2	54.3	43.5	48.7	46.9	51.4	6.38	0.81	0.07	0.64
CD5 + MHCII^hi^	18.1	24.3	18.6	27.0	24.2	23.9	4.54	0.65	0.09	0.38
CD8	7.0	7.9	7.2	6.9	7.5	8.1	1.46	0.80	0.67	0.84
CD45RO + CD8	18.0	17.6	14.0	18.1	13.5	14.6	7.17	0.75	0.70	0.91
NK	5.6	9.9	6.4	9.6	7.3	9.9	3.01	0.91	0.07	0.93
CD45RO + NK	84.2	82.2	87.7	78.5	89.1	91.6	4.56	0.06	0.30	0.26
GD	8.1	10.4	8.8	7.3	8.3	12.7	2.58	0.46	0.27	0.33
CD45RO + GD	82.1	81.6	75.4	71.3	87.3	89.8	7.12	0.04	0.87	0.83
*pHrodo, MFI* ^$^										
CD14	8,981	7,563	6,635	7,244	6,644	8,383	1,693.2	0.67	0.75	0.52
GRAN	9,620^a^	9,076^a^	6,644^b^	9,156^a^	9,339^a^	9,131^a^	668.3	0.05	0.20	0.04
*DHR, MFI* ^$^										
CD14 to UT	3,613	3,350	4,536	3,478	3,130	3,012	937.1	0.29	0.32	0.75
CD14 to PMA	17,225	9,630	15,869	18,479	12,206	15,659	6,802.9	0.66	0.81	0.32
GRAN to UT	4,811	4,861	4,219	4,524	4,540	5,245	634.5	0.57	0.41	0.80
GRAN to PMA	23,906	18,295	25,172	24,942	22,580	24,370	6,653.0	0.64	0.67	0.61

^*^CON = No supplemental Zn; Zn100 = 100 mg Zn/kg DM supplemental; Zn150 = 150 mg Zn/kg DM supplemental; Zn100 and Zn150 supplemented as ZnSO_4_.

^†^All steers were implanted with Component E-S with Tylan (Elanco Animal Health, Greenfield, IN) on day 0. Implant abscess status was determined by a trained evaluator (Elanco Animal Health) on day 16.

^‡^Lower n due to errors with flow cytometry analysis.

^||^CD 21+, CD5+, CD8, NK, and GD cells are % of total population. MHCII^hi^ and CD45RO + are % within the cell type population with those markers.

^$^Mean fluorescence intensity; data normalized using log transformation and back transformed for data presentation.

^a,b^within row and variable, unlike superscripts, indicate differences between means *P* ≤ 0.05 based on pairwise comparisons.

**Table 5.** Effects of dietary Zn concentration and ear implant abscess on immune cell populations and immune response after 56 d

Dietary treatments^*^	CON		Zn100		Zn150			*P* values		
Implant abscess^†^	No	Yes	No	Yes	No	Yes	SEM	Dietary TRT	Abscess	TRT × abscess
*n* (steers)	8	10	5	13	7	11				
*Cell populations, %* ^‡^										
CD21+	21.4	29.5	24.5	25.3	22.4	26.0	2.17	0.75	0.004	0.11
CD21 + MHCII+	24.9	28.2	24.1	22.6	18.5	25.1	3.18	0.15	0.16	0.25
CD5 + MHCII+	15.8	22.0	12.9	15.5	12.9	17.5	2.18	0.02	0.002	0.59
CD4	10.6	9.8	9.9	10.2	10.1	10.8	1.95	0.97	0.97	0.89
CD45RO + CD4	74.5	74.1	86.8	71.9	76.8	75.4	12.90	0.88	0.50	0.74
GD	11.7	10.6	11.2	14.1	14.5	11.9	1.93	0.35	0.85	0.19
CD45RO + GD	48.7	47.4	55.1	41.4	46.1	42.1	8.28	0.77	0.24	0.62
NK	2.5	4.2	2.5	3.7	3.8	3.6	1.00	0.88	0.16	0.47
CD45RO + NK	47.6	40.9	56.5	46.1	48.2	45.7	10.79	0.71	0.35	0.90
*pHrodo, MFI* ^||^										
CD14	10,317	8,230	15,488	9,599	10,048	7,851	4,500.7	0.27	0.21	0.84
GRAN	38,921	26,439	41,936	32,032	42,184	27,165	7,561.1	0.79	0.32	0.83
*DHR, MFI* ^||^										
CD14 to UT	6,122	9,079	7,560	7,616	7,024	7,795	2,116.7	0.99	0.34	0.66
CD14 to PMA	10,317	8,230	15,488	9,599	10,048	7,851	4,500.7	0.34	0.09	0.84
GRAN to UT	5,570	8,672	6,414	5,136	5,809	11,711	4,786.6	0.82	0.51	0.72
GRAN to PMA	38,921	26,439	41,936	32,032	42,184	27,165	7,561.1	0.65	0.002	0.83

^*^CON = No supplemental Zn; Zn100 = 100 mg Zn/kg DM supplemental; Zn150 = 150 mg Zn/kg DM supplemental; Zn100 and Zn150 supplemented as ZnSO_4_.

^†^All steers were implanted with Component E-S with Tylan (Elanco Animal Health, Greenfield, IN) on day 0. Implant abscess status was determined by a trained evaluator (Elanco Animal Health) on day 16.

^‡^CD 21+, CD5+, CD8, NK and GD cells are % of total population. MHCII^hi^ and CD45RO + are % within the cell type population with those markers.

^||^Mean fluorescence intensity; data normalized using log transformation and back transformed for data presentation.

^x,y^within row, treatment means with different superscripts tend to differ 0.05 <* P* ≤ 0.10.

On day 13, there was a main effect for an increased percentage of CD45RO + gamma delta (GD) cells in Zn150 compared to Zn100, with CON being intermediate and not statistically different from either supplemental Zn treatment (TRT *P* = 0.04; Table 4). There was a tendency on day 13 for increased CD45RO + NK cells in Zn150 compared to CON and Zn100 (TRT *P* = 0.06). On day 13, there was also a tendency for increased CD21 + MHCII^hi^ (*P* = 0.10) cells in Zn100 and Zn150 compared to CON. There were no other main effects of TRT for day 13 immune cell phenotypes (*P* ≥ 0.28). On day 13 there was an increased percentage of CD21 + cells in abscessed steers compared to non-abscessed (abscess *P* = 0.03). There was a tendency for increased percentage of CD5 + (*P* = 0.07) and CD5 MHCII^hi^ (*P* = 0.09) cells in abscessed steers compared to non-abscessed steers on day 13. There was a tendency for an increased percentage of NK cells in abscessed steers on day 13 (abscess *P* = 0.07). There were no other main effects of abscess on day 13 (abscess *P* ≥ 0.12).

On day 56, CD5 + MHCII + cells were increased in CON compared to Zn100 and Zn150 (TRT *P* = 0.02; Table 5). There were no other main effects (*P* ≥ 0.15) of dietary TRT for immune cell populations on day 56. There were increased CD21 + (*P* = 0.004) and CD5 MHCII (*P* = 0.002) cells in abscessed steers compared to non-abscessed steers on day 56. There were no other main effects (*P* ≥ 0.16) of abscess on immune cell phenotypes on day 56.

### Functional Assays-Flow Cytometry

On day 13, there was a TRT × abscess effect (*P* = 0.04; Table 4) for Zn100 non-abscessed steers to have decreased MFI in GRAN stain compared to all other treatments. There were no other TRT × abscess effects (*P* ≥ 0.27) on days 13 (Table 4) or 56 (Table 5) for functional flow cytometry assays. There were no main effects of TRT or abscess on day 13 (*P* ≥ 0.29). On day 56, there was increased MFI of GRAN-stained cells from non-abscessed steers compared to abscessed steers (abscess *P* = 0.002; Table 5). There was also a tendency for increased MFI in CD14-stained cells of non-abscessed steers compared to abscessed steers on day 56 (abscess *P* = 0.09). There were no other main effects for flow cytometry functional assays (*P *≥ 0.20).

### Functional Assays—Stimulation

**Figure 2.**
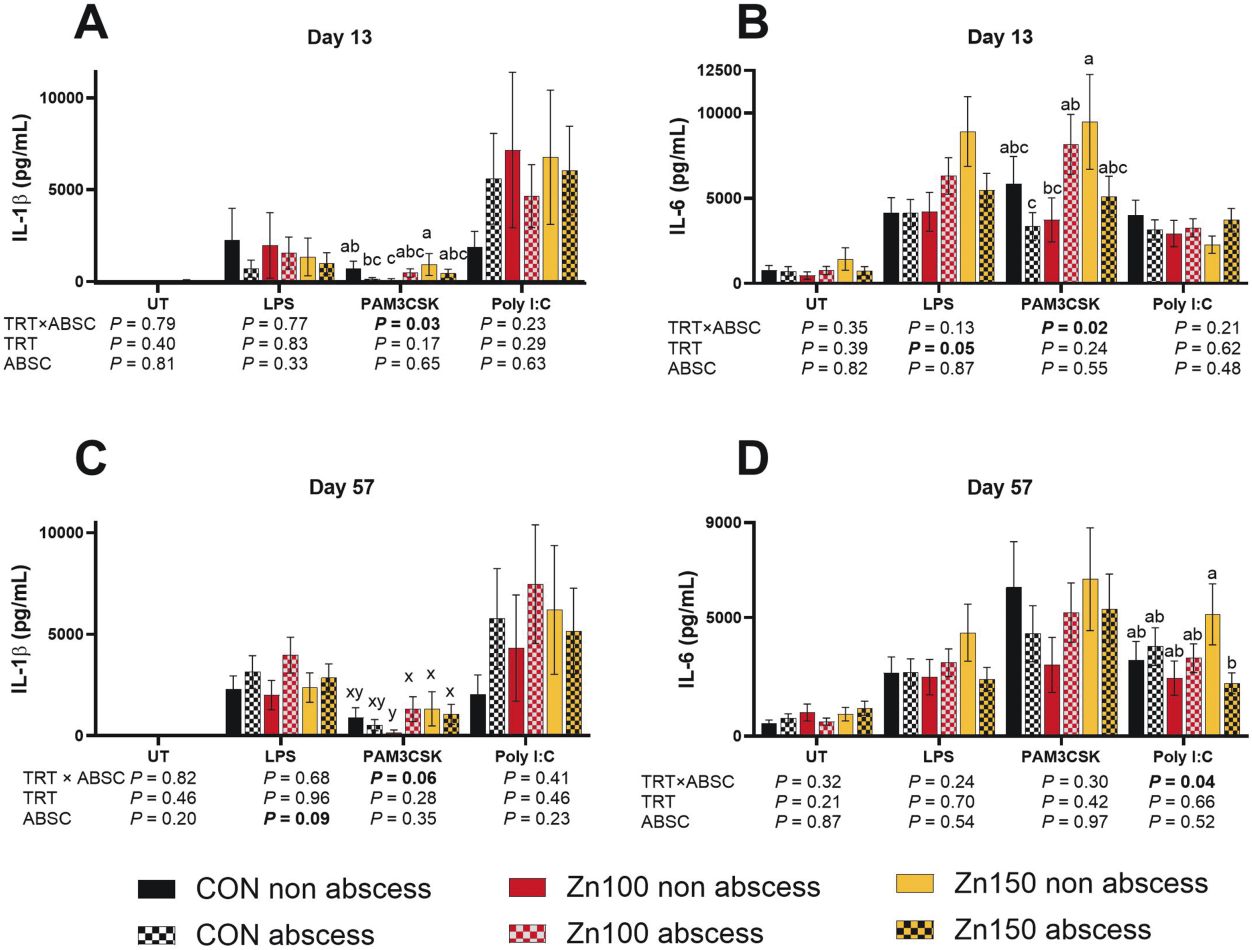
Steers were supplemented with no supplemental Zn (diet analyzed 58 mg Zn/kg DM), 100 mg Zn/kg DM (Zn100), or 150 mg Zn/kg DM (Zn150). All steers were implanted with a Component E-S with Tylan implant (Elanco Animal Health) and dietary treatments started on day 0. All steers were evaluated for implant abscesses (ABSC) on day 16 by a trained evaluator (Elanco Animal Health). Peripheral blood mononuclear cells were plated and incubated with untreated (UT), LPS, PAM3CK, and Poly I:C for 48 h. Cell supernatants were collected and analyzed for IL-1β andIL-6 concentration using ELISA. Within a panel and a stimulant, unlike superscripts indicate a difference in treatments (TRT × absc; *P* ≤ 0.05) or tendency for differences (xyz; *P* ≤ 0.10). (A) On day 13, IL-1β production after stimulation with PAM3CSK was increased in Zn150 non-abscessed steers compared to Zn100 non-abscessed and CON abscessed steers (TRT × abscess *P* = 0.03.) (B) On day 13, IL-6 production after stimulation with PAM3CSK was increased in Zn150 non-abscessed steers compared to Zn100 non-abscessed and CON abscessed (TRT × abscess *P* = 0.02.) After stimulation with LPS, IL-6 production increased in Zn150 steers compared to CON steers (TRT *P* = 0.05.) (C) On day 57, after stimulation with PAM3CSK Zn100 abscess, Zn150 abscess, and non-abscess tended to have greater IL-1β than Zn100 non-abscess (TRT × abscess *P* = 0.06.) After stimulation with LPS, steers with abscesses tended to have greater IL-1β production than steers without abscesses (abscess *P* = 0.09.) (D) On day 57, after stimulation with Poly I:C, Zn150 non-abscess was greater than Zn150 abscess (TRT × abscess *P* = 0.04.)

Cytokines IL-6 and IL-1β were measured after stimulation with toll-like receptor (TLR) agonists. On day 13, there was a TRT × abscess effect (*P* ≤ 0.03; Figure 2) after stimulation with PAM3CSK for both IL-6 and IL-. For IL-6, Zn150 non-abscessed was increased compared to CON abscess and Zn100 non-abscess, Zn100 abscess was also increased compared to CON abscess. For IL-1β, Zn150 non-abscess was increased compared to Zn100 non-abscess and Zn100 abscess, all other treatments were not different. On day 13, after stimulation with LPS, Zn150 increased IL-6 in Zn150 compared to CON (TRT *P* = 0.05).

On day 56, after stimulation with Poly I:C, IL-6 was increased in Zn150 non-abscess compared to Zn150 abscess (TRT × abscess *P* = 0.04), all other treatments were not different. There was a tendency for increased IL-1β after PAM3CSK stimulation on day 56 where Zn100 abscess, Zn150 non-abscess, and abscess were increased compared to Zn100 non-abscess (TRT × abscess *P* = 0.06). There were no other TRT × abscess effects (TRT × abscess *P* ≥ 0.13). On day 56, there was a tendency for increased IL-1β after stimulation with LPS in abscessed steers compared to non-abscessed steers (abscess *P* = 0.09).

## Discussion

Increasing supplemental Zn in cattle diets has grown in interest, given the roles of Zn in growth and immune function. In the current study, supplementing 100 or 150 mg Zn/kg DM from ZnSO_4_ alone did not affect steer BW and overall (day 0 to 57) ADG, DMI or G:F, likely because the control diet, at 58 mg Zn/kg DM exceeded NASEM (2016) recommendations of 30 mg Zn/kg DM. Kegley et al. (2001) observed increased growth performance with Zn supplementation (360 mg Zn/d) to a control diet of 38 mg Zn/kg DM. Smerchek et al. (2023) evaluated different sources of Zn supplementation (103 to 135 mg Zn/kg DM) in growing cattle while also investigating the effects of increased supplementation of Zn (additional 1,000 mg Zn/ steer daily) for the first 14 d of a 42 d study. While growth performance was not affected in the first 14 d, steers fed greater concentrations of Zn had increased feed efficiency for the period after increased supplementation (Smerchek et al., 2023).

This study is unique in that ear abscesses occurred at the site of implant insertion in approximately 60% of the steers, allowing us the opportunity to explore the effects of dietary Zn supplementation and abscesses on immune phenotype and growth. Together, supplemental Zn and implant abscesses tended to alter the ADG and G:F of steers on days 14 to 28 of the study. For ADG, Zn150 steers with abscesses tended to have greater ADG compared to CON and Zn100 steers with abscesses. Feed efficiency was greatest in Zn150 steers with abscesses and tended to be greater than CON with abscesses. Although plasma Zn is considered a poor biomarker for Zn status (Kincaid, 1999), it is interesting to consider the increased ADG and G:F may be driven by a tendency for increased plasma Zn in Zn150 abscessed steers in comparison to the CON abscessed steers. Although not immunocompromised, Messersmith (2021) similarly noted increased growth performance when plasma Zn was increased when cattle were fed 150 mg Zn/kg DM.

Considering implant abscesses alone, steers with an implant abscess tended to have lesser overall (days 0 to 57) ADG and feed efficiency compared to non-abscessed steers. Decreased growth performance with implant abscess could be due to energy shifting towards an immune response (Kvidera et al., 2016) or lack of anabolic stimulus due to the implant being missing. Berry et al. (2000) also noted decreased ADG in cattle with implant abnormalities, though this difference did not affect HCW when compared to cattle with non-abscessed implant sites. Although we did not differentiate between steers with abscesses and steers with missing implants due to abscesses, it is possible that the decrease in performance with abscesses is due to a partial or entirely missing implant in the current study. The lost performance in steers with an abscess at the implant site reinforces the importance of sterile techniques during the implant insertion process.

As part of the adaptive branch of the immune system, B cells respond to infections through antibody production and antigen presentation. The marker cluster of differentiation 21 (CD21) is expressed on mature B cells forming a co-receptor to enhance antigen response (Cherukuri et al., 2001). In the current study, circulating CD21 + B cells were increased on day 13 in steers with abscesses. The marker CD5 on B cells enhances cell receptor signaling, increasing the ability of that B cell to respond to a pathogen, and major histocompatibility complex II (MHCII) is an antigen-presenting molecule. Both CD5 + and CD5 + MHCII^hi^ B cells tended to be increased in abscessed steers compared to non-abscessed. Since these steers had an immune response occurring at the abscess site, the increase in circulating mature B cells, CD5, and MHCII^hi^ cells compared to steers without the immune response, which may result in an increased response time to antigens. This could also be evidence of systemic effects beyond the site of infection.

Dietary Zn treatments altered the percent of activated NK and GD cells with Zn150 steers having a greater percentage of circulating CD45RO + NK and GD cells on day 13. These cells are responsible for recognizing and starting an immune response to infected and abnormal cells (Vivier et al., 2004; Vantourout and Hayday, 2013). Smerchek et al. (2023) also observed changes in CD8 + NK cells after 14 d of increased Zn supplementation, where steers who received increased Zn had an increased percentage of CD16 + cells and a decreased percentage of CD45RO + cells compared to treatments with lower Zn supplementation. In human PBMCs, cells cultured with Zn (60 µM) had increased NK cells compared to cells cultured in an environment without Zn supplementation (Metz et al., 2007). In humans after oral supplementation of Zn, NK cells increased in lytic activity when plasma Zn also increased (Mariani et al., 2008). The marker CD45RO + serves as a marker of activation where cells have greater cytokine responses to stimuli in comparison to the same cell type without CD45RO (Bembridge et al., 1995). In the current study, there was a tendency to have increased CD45RO + GD and NK cells in Zn150 steers compared to CON. It is possible that increased Zn supplementation can alter the activation of these cells which potentially could lead to increased response to pathogen, since both GD and NK cells are innate-like lymphocytes (Van Kaer et al., 2022). In Smerchek et al.(2023) the same marker of activation was measured on CD4 and CD8 T cells, but there was no difference in the % CD45RO + cells based on source or concentration of Zn supplemented. In humans, there was no change in circulating CD45RO + CD3 T cells after 22 wk in supplemented (30 mg Zn/d) compared to not supplemented men (Bonham et al., 2003). It is not clear how Zn supplementation in the current study altered the activation status of cells.

Phagocytosis and oxidative burst are innate immune functions that contribute to pathogen killing by engulfing pathogens and producing substances to neutralize them. The pHrodo and DHR assays assess the phagocytic and oxidative burst ability, respectively, of cells using pH-sensitive fluorescent dyes. The mean fluorescence index (MFI) indicates the “brightness” of the cells, therefore indicating greater phagocytosis or oxidative burst. The current study had a treatment-by-abscess effect on day 13 for GRAN phagocytosis, driven by the decreased MFI in Zn100 steers without abscesses, but it is unclear why these steers had lower phagocytosis compared to all other steers. Few other differences were noted for this assay, suggesting the plasma Zn noted across treatments was sufficient to support this immunological action. However, others have shown the importance of Zn in these activities in vitro (DeCoursey et al., 2003), in humans (Mehta et al., 2013), and in pigs (Kloubert et al., 2018).

Cytokines are an integral part of the immune response, playing roles in activation, migration, and maintaining responses in the immune system. This study investigated the production of cytokines interleukin 6 (IL-6) and interleukin 1β (IL1β) by cells isolated from blood collected from sampling steers that were stimulated with lipopolysaccharide (LPS; a TLR 4 agonist), Pam3CysSerLys4 (PAM3CSK4; a TLR2/1 agonist), and Polyinosinic:polycytidylic acid (Poly I:C; a TLR3 agonist).

In this study, these functional immune assays were minimally affected by dietary Zn alone, on day 13 there was increased IL-6 production in Zn150 steers compared to CON steers. Studies have documented increased cytokine production with additional Zn in simulated cells in culture (Scuderi, 1990). Increased supplementation in pigs has also altered the cytokine, IL-2, response when stimulated with phorbol myristate acetate and Calcimycin (Kloubert et al., 2018).

However, there were several interactions between dietary Zn treatment and abscess presence for IL-6 and IL-1β production. On day 13, after stimulation with PAM3CSK, Zn150 steers without abscesses had the greatest cytokine (IL-6 and IL-1β) production, and were higher than CON abscessed steers and Zn100 steers without abscesses. On day 58, within Zn150, abscessed steers had lower IL-6 production after stimulation with Poly I:C. However, after stimulation with LPS, abscessed steers tended to have increased IL-1β production on day 58. In studies with respiratory disease-challenged animals, production of IL-6 and IL-1β in blood after ex vivo stimulation decreases after infection when compared to the same assay before the disease challenge (Mahmoud et al., 2020; Maina et al., 2024). Due to increased stimulation from infection, TLR response may be decreased due to upregulation of TLR negative regulators (Lang and Mansell, 2007), which may explain the lower cytokine production in abscessed steers. Also, on day 58, after stimulation with LPS, abscessed steers tended to have increased IL-1β production, which could indicate a greater response to pathogen-associated signals. This could suggest systemic and lasting effects of abscesses.

In addition to the altered responses to in vitro immune assays, plasma concentrations of Zn, Fe, and Cu over time can show nutritional immunity. During infection or illness, cytokine signaling upregulates the transporters and storage proteins for Zn, Fe, and other micronutrients to decrease their availability to pathogens (Hood and Skaar, 2012). In the current study, CON steers with abscesses seem to undergo nutritional immunity from days 13 to 28 when plasma Zn decreases, while all other treatments have the same or increased concentration of Zn. Abscessed steers in general also decrease plasma Fe from days 13 to 28, further suggesting systemic and lasting effects of implant abscesses.

Although the development of implant abscesses was an unintended event in this study, the results clearly demonstrate the negative impact of these abscesses on growth performance and immune system function in cattle. This finding underscores the importance of employing proper implantation techniques to minimize the risk of abscess formation. The interactions observed between abscess state and dietary Zn suggest Zn requirements may be influenced by immune challenges, such as infections. Further research is necessary to better understand how Zn requirements are affected by these challenges.

In this study, abscesses had a more significant effect on B cell populations, while Zn supplementation primarily impacted innate-like cells, such as NK and GD T cells. Additional studies are required to gain a deeper understanding of how Zn supplementation in cattle influences circulating immune cell populations and to explore the potential benefits of Zn supplementation in mitigating the negative effects of implant abscesses on growth performance and immune function.
